# Practicality and potential restrictions of unresectable hepatocellular carcinoma prognostic index

**DOI:** 10.1002/hep4.2093

**Published:** 2022-09-20

**Authors:** Coskun Ozer Demirtas, Gabrielle Ricco, Osman Cavit Ozdogan, Feyyaz Baltacioglu, Tunc Ones, Perran Fulden Yumuk, Ender Dulundu, Sinan Uzun, Pierro Colombatto, Filippo Oliveri, Maurizia Rosanna Brunetto, Feyza Gunduz

**Affiliations:** ^1^ Marmara University School of Medicine Division of Gastroenterology and Hepatology Istanbul Turkey; ^2^ Hepatology Unit Pisa University Hospital Pisa Italy; ^3^ Biostructure and Bio‐imaging Institute of National Research Council of Italy Naples Italy; ^4^ Marmara University School of Medicine Department of Radiology Istanbul Turkey; ^5^ Department of Nuclear Medicine Marmara University School of Medicine Istanbul Turkey; ^6^ Marmara University School of Medicine Division of Medical Oncology Istanbul Turkey; ^7^ Department of General Surgery Marmara University School of Medicine Istanbul Turkey; ^8^ Marmara University School of Medicine Department of Medical Biostatistics Istanbul Turkey; ^9^ Department of Clinical and Experimental Medicine Pisa University Pisa Italy

We wish to thank the authors for their comments regarding the recently proposed hepatocellular carcinoma (HCC) scoring system by our group, namely, the unresectable HCC prognostic index (UHPI). The aim of our work was to derive a simple, user‐friendly prognostic tool to be used in every day clinical practice in patients with unresectable HCC. We agree with Adhoute et al. that point‐based scores are generally less powerful than continuous variable models; however, the methodological validation of the approach that we used to develop the score showed its robustness. As indicated in the manuscript, we initially derived a scoring system by assigning exact points for each covariate in proportion to the beta coefficients in the final multivariable model. Then, these covariates were standardized by dividing the smallest coefficient and then rounding to allow simple calculation. Subsequent sensitivity analysis verified that the discriminatory power lost in this simplification process was negligible.

As far as it concerns the values identified by the multivariable analysis in the training cohort, we tested them in an independent European validation cohort and obtained comparable results, which confirmed the superior performance of the UHPI score in predicting survival outcomes.

We proposed the UHPI score as a prognostic classification of patients with unresectable HCC to better stratify their probability of survival. Consequently, the UHPI score was not designed as an algorithm for therapeutic decision, but it can be used to stratify the patients to be enrolled in clinical trials and to compare those observations with the expected survival in “real life.”

In this regard, we thank to authors for testing the UHPI model in a cohort of patients with unresectable HCC treated with a combination of atezolizumab + bevacizumab. In actuality, it was expected that the treatment would change the prognosis and the tumor parameters. Accordingly, as with all prognostic scores, UHPI aimed to predict the survival of patients with HCC but not the objective response to any treatment. Future studies should be addressed to investigate to which extent the power of the UHPI model and its covariates will change in the immunotherapy era.

## CONFLICT OF INTEREST

PC received grants from Gilead Sciences and AbbVie. MRB consults for Janssen and Roche. She consults, advises, and is on the speakers' bureau for Gilead. She advises and is on the speakers' bureau for AbbVie. She is also on the speakers' bureau for EISAI‐MSD.

